# Utilizing Modular Biobanking Software in Different Types of Biobanking Activities

**DOI:** 10.1089/bio.2022.0076

**Published:** 2022-10-17

**Authors:** Laurent Jacotot, Mike Woodward, Axel de Montalier, Philippe Vaglio

**Affiliations:** Modul-Bio, Marseille, France.

**Keywords:** BIMS, biobank information management system, biobank software, LIMS, MBioLIMS BioBanking, Modul-Bio, interoperability

## Abstract

Biobanking defines all activities linked to bioresource management—whether of human, animal, microbial, or environmental origin—which means that any biobank information management system should take into account the multistep life cycle of the samples: from acquisition, through preparation, storage, to distribution to the end users (medical or research teams). Different types of biobanks can use diverse approaches, making it difficult to find software that can handle all types of scenarios. Modul-Bio has developed MBioLIMS BioBanking^®^, a software dedicated to biobanking, as a modular solution so that our various clients can access the functionalities and scale in a system to match their needs. These projects range from biobanks setup and managed by academic institutions, hospitals, and private companies to small and large clinical trials across different countries, as well as to whole campus or organization solutions for multiple biorepositories. Each solution differs in size, requirements, and number of users, from small biobanks with a few members of staff accessing the software to large operations with multiple sites that can collect and ship samples to a centralized site. This article explores different projects that use Modul-Bio's software in a myriad of ways to manage the complete life cycle of biospecimens and associated data.

## Introduction

Alaboratory information management system (LIMS) specifically designed for biobanking is essential for the management and organization of the complete life cycle of biological samples and their associated data. LIMS generally refers to a system for a wide range of laboratories, whereas a biobank information management system (BIMS) is designed for biobanks. Each biobank may have specific needs and functionalities from a system that fits their biobanking model and day-to-day operations.

Although they share many common characteristics, biobanking projects are often very specific and personalized. It is important to be able to rely on a software application that can provide functionality and solutions to each project's key requirements with built-in flexibility and interoperability.

The benefits of a successful BIMS implementation are many because it greatly improves operations, standardizes data collection, and optimizes sample information and management while complying with standards and regulations.

Some examples of successful implementations include single biobanks such as the Pasteur Institute or University of New Mexico to multicountry clinical trials such as global platform for the prevention of autoimmune diabetes (GPPAD) and organizations-wide enterprise models. These are discussed in the following cases in this article.

Biobanks have been using commercial LIMS, custom in-house databases or spreadsheets for many years, but systems have evolved, and are still evolving to be products for this particular field of biobanking, rather than a generic LIMS. Such software has had to evolve to be sustainable and scalable as biobanks store more samples and more data. In addition, there has been more demand for cloud-based options, and data centers, in particular geographical locations, for ease of deployment when a single instance of the software is used by multiple collecting centers.

For the past two decades, the software available to biobanks has changed. Software started mainly as tube-centric for freezer management and has become patient-centric software such as MBioLIMS BioBanking^®^ helping scientists and researchers toward precision medicine, whereas an alternate approach taken by some other software companies is providing adapted biobanking software based on generic LIMS.

Specifically, for BIMS, functionalities have evolved during the course of many years to manage much more information such as patient clinical information, consent, analytical results, allowing different types of sample collections (longitudinal studies, cohorts, and clinical trials), batch work rather than small numbers of samples, as well as integration with new and changing robotics or other medical software.

Modul-Bio has been supplying software for biobanks, biorepositories, and clinical trials for >18 years and created MBioLIMS BioBanking software. This software was developed using Java and runs on a Tomcat Application server; the underlying database is Oracle and it can be deployed either on premises or in the cloud. The interface uses the latest technologies to ensure a smooth and most agreeable user experience with the JavaScript framework Vue.js.

MBioLIMS has added many modular options including in the latest version a workflow module to guide users through processes required and to minimize the risk of human error.

Over this time, the application has grown with biobanking and created developments, most of the time client driven, and always supporting the specific needs of biobanking.

## Case 1: Single Biobank Hospital

In this case, the most common software requirement is a centralized system that manages one biobank location with no additional sites or collecting centers. The hospital itself is the main provider of the samples.

Based on their profiles/credentials, users harness the MBioLIMS BioBanking core modules and the requirements to receive samples in the biobank, store, and add associated data. This information is then available for requests for potential use at a later stage.

Core functionalities include reception and collection of samples, plus storage and processing. This provides most biobanks with the ability to manage their patients, samples, sample data, and clinical data, and perform the tasks needed on a daily basis. As things change, more modules can always be added that are not considered core, but more specific such as robotic integration and temperature tracking.

Samples are received at the biobank from other services, collecting the samples within the hospital; then, they are recorded and entered into the system along with patient information. The BIMS can also record patient information if required, which is captured according to the study in which the patient is included and to collect patient consent.

Unique barcodes are printed for each primary biospecimen container, as well as for each potential aliquot or section and derivatives.

Sample storage management is a critical point for any biobank. It should be designed to recreate the physical storage of the biobank. Storage optimization and actions can be greatly enhanced by defining rules around the type of sample or study for each storage container, and sample types from a study can, therefore, have some reserved storage locations.

**FIG. 1.**
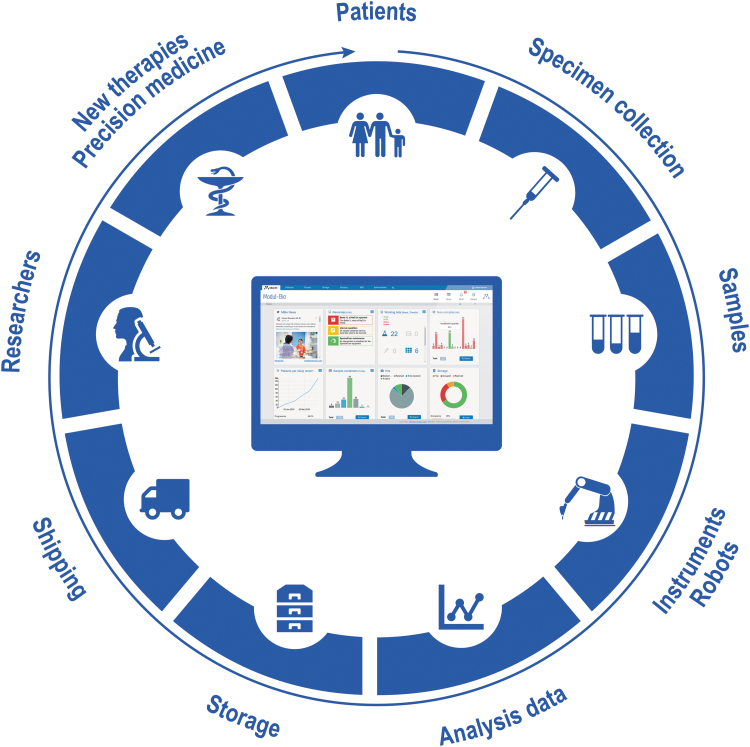
Sample lifecycle in a human biobank.

In this case, samples are registered upon reception and their location history and usage are logged throughout their lifecycle (Fig. 1). This biobank provides reportable information on the storage and movement of all biospecimens.

When samples are requested for research use, their shipment to requestors and their usage are also recorded.

The needs of this biobank were matched to the functionalities already provided in MBioLIMS BioBanking. The successful deployment of the software has been made possible thanks to the biobank staff involved in the design of the parameters needed in the system, including setup of nomenclature, sample types, and processes needed with standard operating procedures (SOPs).

The software saves time for users in performing these actions with intuitiveness, including features such as samples or patients grouped in worklists and the ability to process samples in batches, while all actions are tracked in the software. For example, the aliquoting process in another system at that time required up to 16 clicks, which is reduced to 2 clicks in MBioLIMS and to a single click using the workflow module. These are key elements just to name a few.

Activity reports and statistics regarding reception, sample collection, sample preparation, storage, and shipment can be generated for an entire day, week, month, or year.

To provide a connection to the hospital information system (HIS), a connection using Health Level Seven (HL7), which refers to a set of international standards for transfer of clinical and administrative data between software applications, is very often established with the biobanking software, which allows an automated data transfer of personal health information (PHI) from the HIS to the biobanking software according to the hospital patient ID entered. To keep both systems synchronized and data consistent, updates performed on a patient record in the HIS should be automatically executed and transposed in the biobanking software.

The system is highly secure, and PHI access is only accessible to users with appropriate authorization, ensuring least privilege access control. A key requirement is also that when a patient opts out of a study, the status of the consent will be updated (withdrawal of consent), which could lead to the request of destruction of the patients' samples, including any derivatives. Karubiotec™ the Human Biobank of Guadeloupe (French West Indies) is one of many examples of this first case of implementation.^1^

## Case 2: Research Institute and Academic Center Biobank

### Research institute biobank

The Institut Pasteur (France) research institute biobank is a relevant example for this type of project.

In this project, an HL7 connection was not required, but the main processes were similar to those of a single academic hospital biobank. The main difference is that the samples are often received from outside collecting centers in batches.

The Clinical Investigation and Access to Biological Resources platform of Institut Pasteur provides access to human bioresources for academic and private research teams worldwide, especially in the fields of infection, immunity, and neuroscience.^2^ Human quality-controlled duly annotated samples (mainly whole-blood derived products, but also stool, urine, saliva, swabs, etc.) from both healthy and diseased cohorts with open regulated access are available upon request.

Biospecimens can either be collected from healthy volunteers or patients directly at Institut Pasteur facilities (or at Pasteur Medical Centre) or from collaborating national hospitals or centers worldwide. During the COVID-19 pandemic, the reception of large numbers of biospecimens daily had to be registered in the software using batch sample imports that created multiple records such as patient records, study enrollment, sample collection, sample types, quantity, and storage box position. This specific function has proven instrumental in such exceptional circumstances. Batch uploading large amounts of data did not require additional training as it used the built-in import functionality included with MBioLIMS BioBanking. In terms of scalability, the software is running on a virtual server, therefore, compatible with many platforms, making it easy to manage scalability.

In this case, the software must have additional traceability for the shipping of the sample to the biobank. The capacity to record thousands of samples in a batch is of paramount importance. The software should be capable of handling minimal patient data.

The fine management of specific research projects with, for example, study descriptions and the possibility of allocating specific patients—and their bioresources—to specific studies is also crucial.

### Academic center biobank

Individual biobanks and projects within a university campus often use the system for their own samples, whereas other banks in the same academic institution manage their biospecimens with a different product.

The University of New Mexico Human Tissue Repository (USA) required a system for some integral functionalities to manage their samples.^3^

One of the main requirements was to support the many types of samples and containers in which they were stored. This includes the use of tubes, blocks, slides, and microplates. The management of the collected samples and their ability to be used to form plates and racks within the system was integral.

Considering that new sample types may be worked with in the future, the ability to implement and manage them in the software as they occur is an important element of planning for the repository's future.

Managing the different collections and receptions of samples from multiple sources and facilitating the interaction between users to share sample lists and work with the specimens in the system is one of the desired functionalities.

## Case 3: Multiteam Biobank Within the Same Institution

In this case, multiple teams or laboratories within the same organization use a single piece of software for the management of their biospecimen (i.e., a large hospital with multiple departments, research units, and facilities or a diagnostics company with multiple teams).

Compartmentalization between different departments belonging to the same institution is a key point in multiteam management functionality.

In this case, patient information is shared such that one patient can have samples collected by different departments, such as pathology and hematology, or because there could be multiple pathologies involving this patient in different studies. This means that a biopsy may be collected by the pathology department while bloods are drawn by the hematology department. The different paths for the samples of storage, transport, users processing them, and so on are annotated in the software.

Many different departments of Gustave Roussy, the largest European cancer center, use the MBioLIMS BioBanking software to manage the data as it is used by different teams, such as pathology or hematology, and for different uses, such as clinical trials or cohorts.

Each department can manage the enrollment of a patient in a study, as well as their biospecimens and associated data. Each department can also access samples managed by another department in read mode only if they have been authorized in the administration.

This is particularly useful when researchers need different kinds of materials from the same patient stored in multiple departments, that is, to procure some blood samples associated with biopsies from the same patients stored and managed by two different departments, such as hematology and pathology.

Similar environments have been implemented for diagnostics and biotech companies, which have multiple teams and a centralized biorepository. Using software with compartmentalized access for the management of their bioresources, requests for samples by a team can be addressed to the biorepository which can then retrieve, prepare requested samples, and provide them to the requestor team by using internal transfer of samples.

## Case 4: Multisite (Distant Sites) National Cohort

In this case, client administrators can define user roles and profiles in the system, provide software access to all of them when trained, and specify the data they can access and modify, and the functionalities they are provided with to tailor the required access and experience for different staff (e.g., clinical and biological resources centers [BRC]—staff) and levels.

Regardless of the requirement for software access, compartmentalization options can provide distinct segregated access for as many sites and teams as needed, in addition to a centralized control (administration profile) for the main center of operations to oversee and manage multiple inclusions and collecting centers in real time.

Even in the most complex setup, each group of users can only work with data and menu options that are relevant to them. While a supervising team may see all data across all sites, the general users have an intuitive interface designed via user privileges that allow them to work only with their patients and biospecimens.

The software has powerful functionalities that can be set up but not complex to use. Indeed, all the functionalities configured guarantee ease of use of the patients and samples management, whatever the size of the cohort. The compartmentalization implementation ensures adequate confidentiality and helps in meeting the general data protection regulation prerequisites related to patient PHI. No detrimental impact or slowdowns have been recorded in the CRYOSTEM cohort management since its creation. On the contrary, it offers a global supervision for the administrators while controlling the operations in each center involved in the network.

This could also be a simple scenario of a single study, such as a national cohort, with data and parameters designed to match the information collected. Some biobanks require more versatile systems with multiple sites and a growing number of studies.

Collecting sites were configured to have access to the system and to capture biological, clinical, and demographic data upon sample collection. For each collected period, a data time point was created in the software, providing integral information that groups and annotates samples taken at the same time. Consequently, the system can be used in longitudinal studies and clinical trials conducted at several collection sites. The time point functionality is particularly important for this client. For example, any treatment that a patient receives at the time of sampling can be collected.

Once samples are collected, the software records the shipment of samples from collection sites to the central biobank or the associated BRC for processing and storage, with every step and process related to the lifecycle of the sample. One important factor for the client is to be informed not only of patient inclusion and sampling, but also of the sample shipment and reception through a regular record.

The same feature can be used to send a backup of collections to mirror the biobank samples.

This standardization, completed by a setup of harmonized procedures, ensures better quality of the biological resources and provides prerequisites involved in quality management system certification or accreditation of a multisite network, including collecting centers and laboratories ensuring the cohort constitution.

In this case, the objective of the software is to make multiple sites function uniquely.

An example of such a cohort is CRYOSTEM, the French cohort focused on allogeneic hematopoietic stem cell transplantation complications, including graft-versus-host disease (www.cryostem.org).^4^ The CRYOSTEM cohort is unique in Europe in this regard. In 9 years, CRYOSTEM built a national network involving 36 transplant units and 28 BRCs, which collaborated to set up a collection of >200,000 samples (dried pellets, plasma, and viable cells in dimethyl sulfoxide) derived from blood samples. This network represents >200 people who routinely use MBioLIMS CRYOSTEM to implement the collection.

Almost 6000 patients are being managed using this software. MBioLIMS BioBanking also enables the management of sample provision to national and international researchers, corresponding to a volume of 8000 samples. Updates were performed in the software to follow cohort evolution. For example, a specific module has been added to manage the critical reagent provision and use by BRCs for sample treatment. Moreover, the software enables document management (consent, procedures, and operational documents) by simplifying access to the staff involved in the network.

Another example is the CRYO-LEA cohort, a national cohort focused on child and adolescent leukemia.^5^ In 2018, this cohort included >2000 patients. Two types of samples, blood and skin biopsies, were analyzed using the software. The MBioLIMS CRYO-LEA functions to manage its biospecimen collection very closely to that of the CRYOSTEM cohort.

## Case 5: Clinical Trial

In this case, the clinical trial involved multiple strict compartmentalizations between the sites. Each site will not see the patient or sample data from the other sites.

It is possible to create one supervising site that can access all information across all collecting sites, such as in case 4, even if the physical locations of the sites are in different countries. This allows the supervising site to monitor each site independently, for example, to see the rate of the patient inclusion per week/month. New sites can potentially be added at later stage; thus, the system allows the accommodation of the sometimes-asynchronous nature of some clinical trials between different sites located on different countries.

The GPPAD, https://www.gppad.org, is an international network of research and medicine aimed at a better understanding and prevention of type 1 diabetes.^6^ The GPPAD platform was established in 2015 in Germany, the United Kingdom, Sweden, Belgium, and Poland, with the intention of creating an infrastructure to identify newborns at high risk of developing type 1 diabetes to conduct primary prevention trials.^7^ The platform includes clinical sites and a data coordination center equipped with software for data centralization and analysis, which provides a data repository and regulatory and approval procedures.^8^

The software allows the GPPAD platform to efficiently manage patient enrollment and consent. It also enables longitudinal sample collection tracking, shipping, storage, and management of the overall flow of biological samples between GPPAD sites in Europe, the central laboratory in Munich (Germany), the confirmation laboratory in Bristol (UK), and the biobank in Luxembourg City (Luxembourg). The clinical study centers are located in five European countries: Leuven (Belgium), Dresden, Hannover, Munich (Germany), Warsaw (Poland), Malmö (Sweden), Oxford, and Newcastle (UK).

## Case 6: Campus and Institution-Wide Solution

Using an enterprise version of the software allows larger clients to have segregated banks and multiple collection centers.

The University of California San Francisco and other large universities and organizations register many biobanks on the same campus or institution in a unique system. Collecting centers can also be added, even if they are located outside.

Enterprise-level clients have unlimited users and can create and manage various sites. These sites may be collection centers or other laboratories and repositories within the campus or organization. Although each site can be autonomous, there is also the option to work together on studies if required.

At the campus level, this facilitates a way to manage all the different banks and work on a more common working practice and nomenclature, where possible.

In an institution with multiple banks, it may be that several banks use the same system independently, whereas others use other commercial systems, open sources, or Excel. A migration plan for the first bank to use the new system is then implemented and improved with each new bank that joins the fold.

In such large-scale projects, a central client team can manage the overall project goals and set up in terms of configuration, whereas individual banks have their own teams of users and, in some cases, administrators. Therefore, the ability to configure different biobanks by uploading configuration files is of great advantage in terms of time saving, data consistency, and harmonization.

Institutes conducting studies may need to manage different arms for their studies to create subgroups of patients. It is possible to specify the different arms within a study to create a group of patients.

Workflow management that can be defined in the administration based on a specific study or visit, and sample type combination helps guide users on the sample preparation or analysis that needs to be performed, thereby saving time while reducing errors.

With large projects it is also important to consider client staff resources available and staff turnover. An internal team is often assigned to manage the overall internal implementation and serve as a first line support. Training provided by Modul-Bio is key to set up such a group. The client team is, therefore, in a position to train new staff.

IT resources should also be considered from the client side, not only to support installation and operation, but also to facilitate other imports and processes, set up application programming interface use with third-party software and further streamline processes.

Introducing a BIMS in an institution greatly improves traceability, chain of custody leading to high-quality biospecimen management while ensuring compliance with regulations and ethics.

One measure of this is a change in ability, such as the possibility to search and query across multiple sets of banks that previously was not possible as they were using separated systems. This also allows the standardization of many items, including nomenclature, standard practices, and processes. In many cases a key metric is user time, for example, using a BIMS instead of spreadsheets, or being able to perform tasks in large batches rather than sample by sample.

## Conclusion

In conclusion, it is important to consider dedicated biobanking software versus generic software configured for this purpose. The latter has been built up over time with functionalities and requirements from the biobanking sector, such as integration/interfaces with HIS, instruments, robotics, and temperature monitoring systems. This continues to develop, ensuring the needs of the biobanking community are represented in a BIMS.

A limitation of this approach when moving and/or migrating to a BIMS is the quality of the data in legacy systems. A data transfer process can be time consuming, and older data may need to be reviewed, but the benefits of a BIMS with searchable data in the correct format is clear.

In addition, dedicated biobanking software provides a more streamlined data management process, intuitiveness, faster recording, and better decision-making.

The scalability of biobanking software with a wide range of features is key to providing a centralized solution fitted to the numerous biobanking activities that can be performed by the hospital biobank, a national cohort, a clinical trial, or even a network of biobanks campus wide. However, Modul-Bio software can be tailored to the specific needs of clients by sharing functionalities and modules already built for other similar clients and designed for these types of biobanking activities.

The functionality in a system, ease of use, dedicated biobanking modules, and flexibility represent real added value for clients managing smaller or large-scale biobanks. Varied requirements and future changes in workflows must be managed within the system.

For quality management and certification, a BIMS can also be of great use in SOP management, auditing, and sample history. Searchable and reportable data that can be accessed when required to investigate, formulate, and report biobanking operations and specimen use.
